# Conversion of PET Bottle Waste into a Terephthalic Acid-Based Metal-Organic Framework for Removing Plastic Nanoparticles from Water

**DOI:** 10.3390/nano14030257

**Published:** 2024-01-24

**Authors:** Chingakham Chinglenthoiba, Gomathi Mahadevan, Jiawei Zuo, Thiruchelvam Prathyumnan, Suresh Valiyaveettil

**Affiliations:** Department of Chemistry, National University of Singapore, 3 Science Drive 3, Singapore 117543, Singapore

**Keywords:** microplastics, nanoplastics, environmental pollution, metal-organic framework, water purification, polyethylene terephthalate (PET) bottle waste

## Abstract

Micro- and nanoparticles of plastic waste are considered emerging pollutants with significant environmental and health impacts at high concentrations or prolonged exposure time. Here we report the synthesis and characterization of a known metal-organic framework (MOF) using terephthalic acid (TPA) recovered from the hydrolysis of polyethylene terephthalate (PET) bottle waste. This approach adds value to the existing large amounts of bottle waste in the environment. Fully characterized zinc-TPA MOF (MOF-5) was used for the extraction and removal of engineered polyvinyl chloride (PVC) and polymethylmethacrylate (PMMA) nanoparticles from water with a high efficiency of 97% and 95%, respectively. Kinetic and isotherm models for the adsorption of polymer nanoparticles (PNPs) on the MOF surface were investigated to understand the mechanism. The Qmax for PVC and PMMA NPs were recorded as 56.65 mg/g and 33.32 mg/g, respectively. MOF-5 was characterized before and after adsorption of PNPs on the surface of MOF-5 using a range of techniques. After adsorption, the MOF-5 was successfully regenerated and reused for the adsorption and removal of PNPs, showing consistent results for five adsorption cycles with a removal rate of 83–85%. MOF-5 was characterized before and after adsorption of PNPs on the surface using a range of techniques. The MOF-5 with PNPs on the surface was successfully regenerated and reused for the adsorption and removal of polymer nanoparticles, showing consistent results for five extraction cycles. As a proof of concept, MOF-5 was also used to remove plastic particles from commercially available body scrub gel solutions. Such methods and materials are needed to mitigate the health hazards caused by emerging micro- and nanoplastic pollutants in the environment.

## 1. Introduction

Plastics are widely used in many products owing to their low density, easy access in large quantities, high processability, stability, and high versatility in applications. Plastic materials are assimilated into every aspect of our daily life and offer great convenience to human life [1]. In recent decades, an exponential increase in plastic production and usage led to a significant increase in plastic waste generation and high demand for proper waste management. The most common plastics are polyethylene terephthalate (PET), polystyrene (PS), polypropylene (PP), polyethylene (PE), and polyvinyl chloride (PVC) due to their mass production, durability, versatility, and low cost [2]. Large amounts of nonbiodegradable synthetic plastic waste materials are accumulating in the environment and causing significant damage to the ecology [3].

The concentration of micro- and nanoplastic particles in the environment vary widely depending on a range of factors, including location, weather conditions, multiple sources of plastic wastes and the existing methods for waste processing. In addition, the analytical methods used for collection, separation, detection, identification, and quantification of plastic particles also vary significantly [4]. One of the key challenges in understanding the concentrations of such small plastic particles in water is the lack of standard methods for detecting and quantifying these particles. Different studies have used various techniques, such as density separations and filtrations for collecting the particles and microscopic and spectroscopic methods for the detection and identification of the microplastic particles [5,6]. The small size and low density of polymer particles and their ability to be carried long distances by wind or water makes them difficult to track and quantify [7,8]. Despite these challenges, a growing body of research is providing insights into the contamination of the microplastic and nanoplastic particles in water resources and the potential impacts of such particles on ecosystems and human health [9,10,11]. The data collected on plastic particles in the environment are used to mitigate the risks posed by plastic pollution and to develop strategies for reducing plastic particle contamination in the environment [12]. A few reports are available in the literature for the removal of plastic nanoparticles from water [13,14,15].

Compared to the existing water purification technologies, adsorption method is a simple and efficient technology for removing dissolved pollutants from water [16,17,18]. A few adsorbent materials such as cellulose [19] silica [20], zeolite [21], activated carbon [22], biochar [23], metal organic framework (MOF) [24], chitosan [25] and carbon nanotubes [26] were used for water purification. The above-mentioned adsorbent materials showed some disadvantages such as low uptake capacity, low selectivity and poor stability in water. 

MOFs are a class of porous materials synthesized from different metal ions and a series of organic ligands [27,28,29]. Owing to ordered porous structure, enhanced chemical stability, easy processability, high surface area, structural and chemical diversities in surface functional groups [30], MOFs are used for the removal of pollutants from water [31,32,33] gas separation [34], fluoride ion removal [35], and sensors for pollution detection [36]. The high-density active sites in MOFs and ordered pores allow selective recognition and adsorption of target molecules from different media [37]. A recent study showed that MOF different metal ions and terephthalic acid (TPA) was useful towards removing different types of pollutants such as dyes, heavy metals, bisphenol A and pharmaceuticals from water [32,38].

**Scheme 1:**
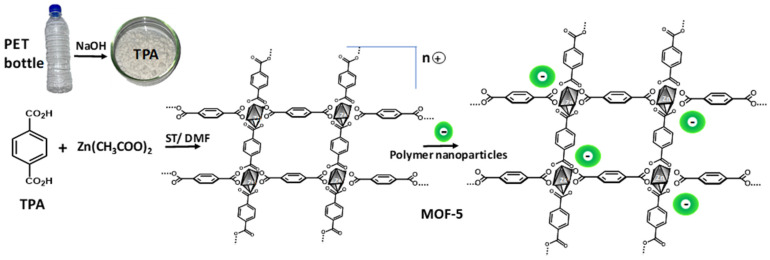
Schematic representation on the preparation of TPA, MOF-5, and extraction of PNPs from water.

Plastic pollution is a critical environmental issue, and the accumulation of nanoplastic particles in wastewater has raised serious concerns about their impact on the ecosystem and human health. In this context, we report the synthesis of metal-organic framework (MOF) using terephthalic acid (TPA) recovered from waste PET bottles via hydrolysis and zinc salts. Such water insoluble MOFs offer high surface charges and increased porosity for the adsorption of a range of pollutants from water. Fully characterized “recycled” MOF-5 was used for removing engineered PMMA and PVC NPs from water. The current study demonstrates a simple strategy for mitigating plastic pollution and value addition to a common plastic waste material, PET. This method is a sustainable, nontoxic and efficient solution for removing polymer nanoparticles (PNPs) from water (Scheme 1). 

## 2. Materials and Methods

### 2.1. Materials

Discarded polyethylene terephthalate (PET) bottle wastes were collected from local canteen and used for preparing terephthalic acid (TPA). Sodium hydroxide (NaOH), sodium dodecyl sulfate (SDS), ethanol, hydrochloric acid (HCl), zinc acetate (Zn(CH3COO)2), tetrahydrofuran (THF), dimethyl formamide (DMF), polyvinyl chloride (PVC, Mw~120,000), and polymethylmethacrylate (PMMA, Mw~350,000), were purchased from Sigma Aldrich. Body gel scrub was purchased from a local market. All chemicals were at the highest purity level (99.99%) available in the market and used as received. Deionized (DI) water was used for preparing the solutions and conducting experiments. 

### 2.2. Conversion of PET into TPA

**Figure 1:**
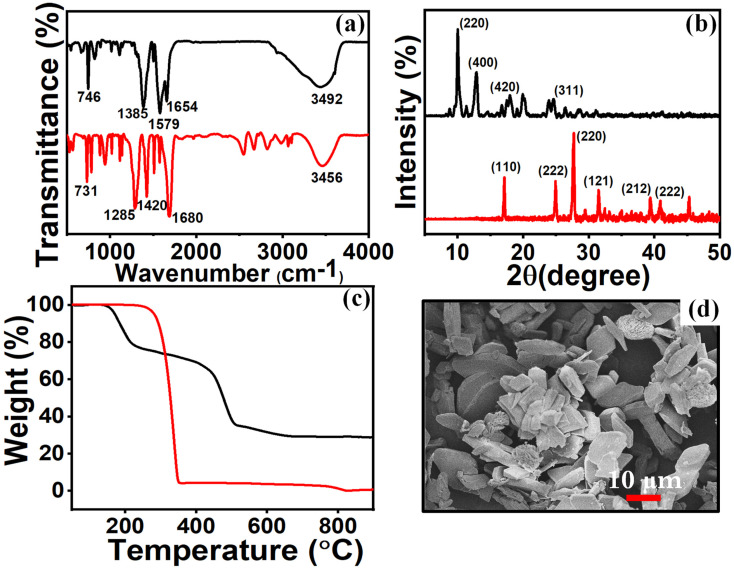
FTIR spectra (**a**), XRD pattern (**b**), TGA (**c**) and SEM (**d**) of freshly prepared TPA (▬) ligand and MOF-5 (▬). FTIR spectra were recorded using KBr as a matrix, powder XRD patterns were collected under ambient conditions, and TGA traces were recorded under an N_2_ atmosphere using a heating rate of 10 °C/min.

A reported procedure was used for the hydrolysis of PET bottle fragments to prepare sufficient amounts of TPA [39]. In brief, the PET bottle wastes were chopped into small pieces (0.5 cm × 0.5 cm) using a scissor and used for the hydrolysis reaction with sodium hydroxide solution. PET bottle pieces (2 g), NaOH (25 g, excess), ethanol (200 mL), and SDS (1 g) were taken in a round bottom flask and refluxed for 4 h. After cooling to room temperature, the mixture was acidified with dil. HCl solution (1 M) to get a pH value of 3. The white precipitate obtained was washed with distilled water (20 mL × 3) and ethanol (20 mL × 2), and dried at 60 °C for 24 h. The obtained TPA powder was further characterized using spectroscopic techniques and compared with the literature data (Figure 1). 

### 2.3. Synthesis of MOF-5

The MOF-5 was prepared using a solvothermal method. Zinc acetate (Zn(CH_3_COO)_2_, 1.32 g, 6.02 mmol) and terephthalic acid (TPA, 0.5 g, 3.01 mmol) were taken in a molar ratio of 2:1 and mixed in DMF (10 mL) to prepare the MOF [40,41]. The acetate ions act as a base to deprotonate TPA and the solvothermal reaction was carried out in a Teflon autoclave reactor at 120 °C for 24 h. The reacted mixture was centrifuged and washed with DMF, and the solid was collected and dried in an oven at 60 °C for 24 h. 

### 2.4. Preparation of Fluorescent Nanoparticles from PVC and PMMA

The efficiency of MOF-5 for removing plastic nanoparticles from water was investigated using fully characterized luminescent polymer nanoparticles (PNPs). Two polymer nanoparticles (PNPs) from common synthetic polymers, such as PVC and PMMA, encapsulated with a fluorescent perylene dye were prepared using a previously reported nanoprecipitation method [42]. The appropriate polymer (400 mg), SDS (10 mg, 4 wt% of polymer), and perylene tetrabutylester dye (10 mg, 4 wt% of polymer) were dissolved in acetone or tetrahydrofuran (50 mL). A fixed volume (5 mL) of the clear solution was quickly added to 50 mL of sterilized water, stirred overnight to allow the slow evaporation of organic solvent, and filtered through a cotton plug to get rid of any large particles or contaminants [16]. All prepared PNPs were characterized using UV-Vis spectroscopy, fluorescence spectroscopy, dynamic light scattering (DLS), and scanning electron microscopy (SEM). Stock solutions of different concentrations of the polymer nanoparticles in water were prepared and used for the extraction experiments.

### 2.5. Batch Adsorption Studies

The adsorption experiments with MOF-5 and PNPs were performed at room temperature. In the batch method, a fixed volume of the aqueous PNP solution (10 mL, 250 ppm) was used for all experiments. The appropriate amount of MOF-5 (25 mg) was added to the PNP solution, and the mixture was shaken on a mechanical shaker for different periods, 5–60 min, under ambient conditions. After adsorption, the mixtures were centrifuged to separate the solid adsorbent, and the supernatant was analyzed using UV-Vis and fluorescence spectroscopic measurements to calculate the residual PNPs left in the supernatant solution. Each adsorption was repeated three times, and an average value was used for calculations. The concentration of PNPs in the solution was determined using a calibration curve, which was prepared using absorption and emission intensities of varying concentrations of standard PNP stock solutions (Figure S1). 

The adsorption efficiencies were calculated using Equation (1) based on the differences between the initial and final concentrations of the fluorescent plastic nanoparticles.

(1) $$\mathrm{Adsorption}\;\mathrm{efficiency}\;(\%)=\frac{\left(C_{0}-C_{1}\right)}{C_{0}}*100\%$$

where C_0_ and C_1_ represent the concentrations of PNP solutions before and after adsorption on MOF-5 adsorbent, respectively.

The kinetic adsorption studies were performed based on time-dependent studies using samples collected at 5 to 60 min.

The adsorption capacity, Q_e_ was calculated from the following equations,

(2) $$\mathrm{Adsorption}\;\mathrm{capacity}\;(Q_{e})=(\frac{C_{0}-C_{e}}{m})*V$$

where C_o_ and C_e_ refer to the initial and the equilibrium concentrations, respectively, of the PNPs in mg/L, V is the volume of the solution (mL), and m is the mass (mg) of the dry adsorbent. All experiments were conducted under ambient conditions to understand the mechanism of adsorption of PNPs on the surface of MOF-5.

### *2.6.* Extraction of Microplastic Particles from Body Scrub Gel Using MOF-5

To demonstrate the efficiency of MOF-5 towards the adsorption of PNPs, a commercial body gel scrub containing micro- and nanoparticles of plastic particles was used. The body gel scrub (1 mL) was diluted with DI water (50 mL) and used for the adsorption experiments water [14,39,43]. A fixed amount of MOF-5 (25 mg) was added to the diluted body gel scrub (10 mL) and shaken for 60 min. The scrub solution was allowed to settle, supernatant solution was separated form the solid particles via filtration. Both the aqueous filtrate and the solid MOF were analyzed to determine the efficiency of adsorption. The solid MOF with plastic particles on the surface was analyzed using FTIR spectroscopy and SEM microscopy. The supernatant solution was analyzed using DLS to check the presence and size distribution of plastic particles.

### 2.7. Regeneration of the MOF-5 after Adsorption

Regeneration of the adsorbent helps to create a more sustainable low-wastewater purification method. Here, the MOF-5 particles (25 mg) with PNPs adsorbed on the surface were agitated with dilute HCl solution (10 mL, 0.001M) using a mechanical shaker at 300 rpm for 60 min. The agitated mixture was centrifuged to separate the supernatant solution from the solid MOF-5. The supernatant solution was collected for UV-Vis spectroscopic analysis, and the data was used to calculate the desorption efficiency. The regenerated MOF-5 was then used to extract PNPs from a fresh solution and calculate the readsorption efficiency. To understand the MOF particles’ regeneration efficiency, desorption/readsorption efficiencies were calculated for five consecutive cycles.

### 2.8. Characterization Methods

The infrared spectra (FTIR) of the samples were recorded in the range of 4000–400 cm^−1^ using a Bruker ALPHA FT-IR spectrophotometer. The concentration of Zn metal in MOF-5 was analyzed using inductively coupled plasma emission spectroscopy (ICP-OES) to establish the structure. X-ray diffraction (XRD) studies were carried out using the powder X-ray diffractometer of the Bruker-AXS D8 DISCOVER instrument (Cu Kα radiation λ = 1.540 Å). The surface structure of MOF-5 was established using a field emission scanning electron microscope (FESEM—FEI Quanta 250F). Thermogravimetric analyses (TGA) were conducted to understand the thermal stability of the adsorbent samples. Quantitative analysis of the extraction of PNPs was carried out using a Shimadzu UV-1601 UV-Vis spectrophotometer and an Agilent Cary Eclipse fluorescence spectrophotometer. The size and surface charges of the prepared PNPs were measured using a Malvern Zeta sizer Nano-ZS90 instrument. BET, pore size, total pore volumes, and Langmuir surface areas of the samples were determined from nitrogen (N_2_) adsorption isotherms using the Quantachrome Autosorb iQ C-XR model to determine the pore size and surface area. The samples were degassed for 12 h at 120 °C before analyzing the sample.

## 3. Results and Discussion

The hydrolysis of the PET is a straightforward reaction leading to solid TPA and the structural characterization was done using FTIR spectroscopy recorded in the range of 4000–500 cm^−1^ (Figure 1a and Figure S2). The spectrum was compared with PET polymer and commercial high-purity TPA samples (Figure S2). The >C=O stretching vibration and C-O vibration of the ester bonds in PET are observed at 1717 and 1244 cm^−1^, respectively. For comparison, the >C=O and C-O stretching vibrations were observed at 1680 and 1285 cm^−1^, which are attributed to the -COOH group in the TPA molecule (Figure S2). The –OH groups of the TPA molecule showed broad peaks around 3456 cm^−1^ (stretching) and 1420 cm^−1^ (bending) vibrations. The peak at 731 cm^−1^ represents the C-H bending vibration of the substituted benzene ring [19,40,44,45]. The peaks at 1654 cm^−1^, 1579 cm^−1^, and 1385 cm^−1^ in the FTIR spectrum of MOF-5 are attributed to the coordination bond of C-O-Zn (Figure 1a) [41]. Several small peaks around 746 cm^−1^ are due to the out-of-plane bending vibrations of C-H bonds characteristic of 1,4-substituted benzene [46].

The structure of the extracted TPA was also characterised using XRD studies and the peak positions in the XRD pattern were compared with the reported values (Figure 1b) [46,47]. The peaks observed on the XRD pattern of TPA powder were indexed as 2θ = 17.15° (110), 24.96° (222), 27.67° (220), 31.57° (121) and 39.44° (212). The *d*-spacing corresponding to each XRD peak were also calculated as 5.16 nm, 3.56 nm, 3.22 nm, 2.83 nm and 2.28 nm using the Scherer equation and agrees with the reported values [47]. 

The synthesized MOF-5 was characterized using XRD data to understand the lattice structure of the material. The observed XRD pattern of the MOF matches with the reported values and the peaks were indexed as 2θ = 9.95° (220), 12.91° (400), 17.97° (420), 19.77° (311), and 23.99° (531) (Figure 1b) [41]. The *d*-spacing calculated for each diffraction peak seen in the XRD pattern using Scherrer equation gave values 8.87 nm (9.95°), 6.84 nm (12.91°), 4.92 nm (17.97°), 4.64 nm (19.77°) and 3.8 nm (23.99°). The reported MOF has the monoclinic crystal lattice with a space group of *P*2_1_/n and the unit cell parameters of *a =* 6.65 Å, *b =* 15.22 Å, and *c =* 12.61 Å [41]. The reported single crystal structure of MOF-5 involves TPA complexes of Zn^2+^ ions, which forms a 3D asymmetric MOF (CCDC 271518). So far, our attempts to grow good quality single crystals from MOF-5 material were unsuccessful. 

The thermal decomposition of the MOF-5 was studied using thermogravimetric analyses (TGA) within a temperature range of 25 °C to 900 °C using a heating rate of 10 °C/min (Figure 1c). The TPA ligand and MOF showed significant differences in thermal degradation patterns [39]. The MOF-5 showed a weight loss at 167 °C (24.9%), which corresponds to the loss of solvent molecules, including the moisture trapped inside the framework, and the second weight loss at 465 °C (42.5%) due to the decomposition of the TPA ligand. A residual mass of 30.6 % was obtained after heating the sample to 900 °C. The elementary analysis data for the as prepared MOF-5 showed 18.97% of zinc metal (Supplementary Materials, Table S1). The difference in residual mass from TGA (30.6%) and ICP data of zinc (18.9 %) is due to the formation of ZnO under high heat or metal content with undecomposed carbon-based materials. The ICP data gives only the amount of Zn present in the digested solution of MOF, whereas, TGA analysis involves thermal degradation of MOF at high temperatures, which leaves only the metal residue and highly stable carbon analogs [48]. The strong coordination between Zn metal ions and TPA enhanced the thermal stability of MOF [40,49,50,51].

The surface morphology of MOF-5 was examined using a scanning electron microscope (SEM), and many thick flat flakes were observed in the micrograph (Figure 1d). The TEM images (Figure S3a) and the elemental mapping of MOF-5 (Figure S3b–d) revealed the presence of zinc and oxygen atoms on the surface of MOF. The SEM micrographs of engineered PVC NPs and PMMA NPs and the size distribution of the nanoparticles are recorded (Figure S4a–c). The SEM images showed spherical morphologies with a dry state diameter of ~95 nm for PMMA NPs and ~75 nm for PVC NPs, which is usually smaller than the hydrated size values obtained from DLS. The average hydrodynamic diameter of the hydrated PVC NPs and PMMA NPs were recorded as ~155 nm and ~189 nm, respectively (Figure S4c). The observed differences in the size of the PNPs obtained from SEM and DLS are consistent with the reported literature [52]. The zeta potentials measured were −21.1 mV, −27.5 mV, and 36.25 mV for PMMA NPs, PVC NPs, and MOF-5, respectively. The mechanism of adsorption depends on the porosity of the MOF-5 and the electrostatic interactions between the adsorbent and adsorbate. The strong positive surface charge of MOF-5 and the negative zeta potential of PNPs are highly desirable for an effective adsorption process. BET traces of the MOF-5 showed the appearance of a type III multilayer adsorption isotherm (Figure S3e). The BET analysis gave a specific surface area of 27 m^2^/g, with the size and volume of pores as 3 nm and 0.3 cc/g, respectively, which are closer to the reported values [53]. The SEM images showed spherical morphologies with a dry state diameter of ~95 nm for PMMA NPs and ~75 nm for PVC NPs, which is usually smaller than the hydrated size values obtained from DLS. The average hydrodynamic diameter of the hydrated PVC NPs and PMMA NPs were recorded as ~155 nm and ~189 nm, respectively, from DLS studies (Figure S4c). The observed differences in the size of the PNPs obtained from SEM and DLS are consistent with the reported literature [52]. The zeta potentials measured were −21.1 mV, −27.5 mV, and 36.25 mV for PMMA NPs, PVC NPs, and MOF-5, respectively. The positive surface charge of MOF-5 and the negative zeta potential of PNPs are highly desirable for an effective adsorption process. BET traces of the MOF-5 showed the appearance of a type III multilayer adsorption isotherm (Figure S3e). The BET analysis gave a specific surface area of 27 m^2^/g, with the size and volume of pores as 3 nm and 0.3 cc/g, respectively, which are closer to the reported values [53].

**Figure 2:**
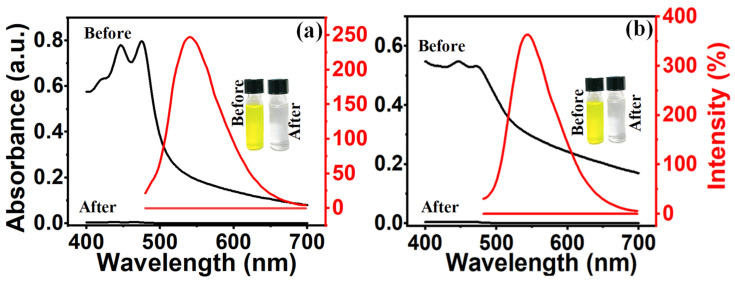
UV-Vis absorption (▬) and fluorescence (▬) spectra of PVC NPs (**a**) and PMMA NPs (**b**) solutions in water before and after adsorption. The lack of absorbance and fluorescence intensities after adsorption show that all PNPs are removed by the MOF-5 from the solution. The concentration of PNPs used was 250 ppm. The optical images of the solutions before and after adsorption experiments are given in the inset. The amount of MOF-5 used was 25 mg in 10 mL PNP solution. The excitation wavelength used for the emission spectrum was 476 nm.

The UV-Vis spectra and fluorescence spectra of PNP solutions in water before and after adsorption on MOF-5 were recorded to track the adsorption of the nanoparticles (Figure 2). An excitation wavelength of 476 nm was used for recording the fluorescence spectrum of the perylene tetraester (PTE) dye encapsulated inside the particles. The inset shows the optical images of the polymer nanoparticle solution (250 ppm) before and after adsorption on the MOF-5 (25 mg), which showed changes in color from pale yellow to a colorless solution after removing the PNPs from water. The UV-Vis spectra of solutions before and after adsorption experiments showed complete removal of PVC NPs and PMMA NPs from the samples after shaking with MOF-5 (Figure 2).

**Figure 3:**
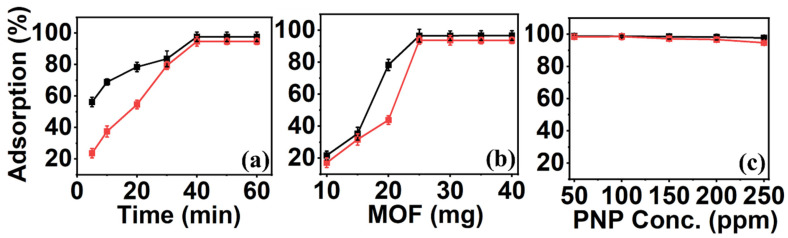
Extraction efficiency of PVC NPs (▬), PMMA NPs (▬) using MOF-5 at variable extraction times (**a**), different MOF loadings (**b**), and concentrations of polymer nanoparticles (**c**). In (**a**), the concentration of PNPs used was 250 ppm and the adsorbent amount 25 mg; in b, the concentration of PNPs was 250 ppm for 60 min of studies, and in c, the amount of MOF was 25 mg in 10 mL solution of PNPs and shaken for 60 min where concentrations of the PNPs varied from 50 to 250 ppm.

A few experimental factors influence the adsorption of PNPs on the surface of MOF-5. To understand the influences of these different parameters on the adsorption process, the experiments with increasing the amounts of MOF, and varying the extraction times at different concentrations of PNPs, were conducted and the results are given in Figure 3.

### 3.1. Effect of Time on Adsorption of PNPs

The extraction time of the PNPs varied from 0 to 60 min to understand the adsorption process (Figure 3a). The adsorption efficiency of the MOF was evaluated after collecting samples at various time intervals (0–60 min). The adsorption of PNPs on MOF-5 showed an adsorption equilibrium within 40 min in the presence of fixed amounts of MOF (25 mg) and PNPs (250 ppm) in solution. The adsorption of the PVC NPs and PMMA NPs on MOF surface increased with an increase in extraction time from 5 min to 40 min. The high removal efficiency observed during the initial period is attributed to the strong electrostatic interaction between the positively charged MOF and the negatively charged PNPs [54,55,56] Also, the initial rate of adsorption of the PVC NPs is higher than the PMMA NPs owing to the differences in zeta potential values. The maximum adsorption percentage was obtained within 30 min with an adsorption efficiency of 97% and 95% for PVC NP and PMMA NP solutions, respectively, calculated based on Equation (1).

### 3.2. Effect of Changes in Concentration of MOF-5

The dosage of the MOF used for the adsorption of PNPs from water also influences the removal efficiency (Figure 3b). The experiments were performed by using the amount of MOF-5 from 5 to 40 mg and a fixed concentration of PMMA and PVC NP solution (10 mL, 250 ppm). The extraction efficiency increased with the amount of MOF used, owing to the number of active surface sites for the adsorption. The percentage removal reached a maximum at an optimum amount (25 mg) of MOF-5, which is sufficient for efficiently removing PNPs from water. Excellent adsorption efficiencies of 97% for PVC NPs and 95% for PMMA NPs were achieved within a short extraction time of 30 min using the MOF adsorbent.

### 3.3. Influence of PNP Concentration on Adsorption Efficiency

Another parameter that influences the extraction efficiency of PNPs is the concentration of the PMMA NPs and PVC NPs (Figure 3c). The effect of the concentration of the PNPs (50–250 ppm) on the extraction efficiency of MOF-5MOF (25 mg) was investigated. The MOF showed an exceptionally high removal efficiency at a concentration of approximately 50 ppm for both PMMA and PVC NPs. The initial adsorption rate was high, almost 100%, at low concentrations of PNPs because of the increased mass transfer from water to the adsorbent surface and the availability of a large number of surface functional group [57].

### 3.4. Kinetic Study on the Adsorption of PNPs on MOF Surface

The pseudo-first-order and pseudo-second-order kinetic studies were performed to understand the adsorption mechanism of PNPs on the surface of MOF-5.

(3) $$\ln\;(Q_{e}\;-Q_{t})=\mathrm{In}\;(Q_{e})-K_{1}t\;\mathrm{Pseudo-first-order}\mathrm{model}$$

(4) $$t/Q_{t}=t/Q_{e}+1/k_{2}Q_{e}^{2}\;\mathrm{Pseudo-first-order}\mathrm{model}$$

where, $Q_{e}$ and $Q_{t}$ represents the quantities of the pollutants adsorbed by the MOF-5 at equilibrium and at time t (min), respectively. The values of $K_{1}\;and\;k_{2}$ represents the rate constants for the adsorptions under different conditions. The collected data were processed and plotted in the Supplementary Materials (Figure S5). The following table summarizes all theoretical and experimental parameters measured during the experiments (Table 1).

**Figure 4:**
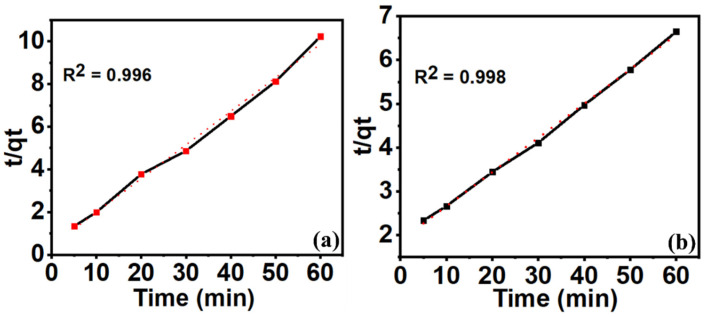
Kinetic studies using pseudo-second-order model for the adsorption of PVC NPs (■, **a**) and PMMA NPs (■, **b**) on MOF-5 from water. The dotted line represents the best fit line.

The pseudo-second-order kinetic model correlated well with experimental data and gave a value of the regression coefficients (R^2^) as 0.99 (close to 1) for both PNPs (Table 1). In addition, the calculated Q_e_ values for the extraction of PNPs from the pseudo-second-order model are comparable to the experimental Q_e_ values obtained. Minimum differences between the calculated and the experimental $Q_{e}$ values were observed. The pseudo-second-order treatment showed the best data agreement as compared to the pseudo-first-order kinetic equation for the adsorption of PNPs on MOF (Figure 4, Figure S5)) [57,58].

### 3.5. Determination of the Mechanism of PNP Adsorption on MOF-5

**Figure 5:**
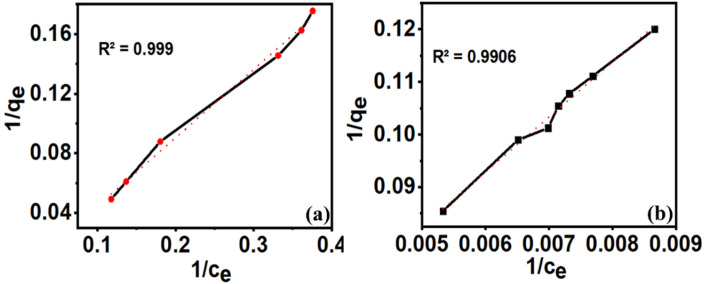
Langmuir isotherm model studies for the adsorption of PVC NPs (●, **a**) and PMMA NPs (■, **b**) on MOF-5 from water. The dotted line represents the best fit line.

The mechanism of adsorption is described by the adsorption isotherm. To assess the relationship between the adsorbate and the adsorbent, various isotherm models such as Langmuir, Freundlich, Temkin, and Dubinin-Radushkevich were employed, and the results are summarized in Table 2, Figure 5 and Figures S6–S8.

Among all the models tested using the adsorption data, the Langmuir isotherm model showed the best correlation, which indicates that the adsorption of PNPs forms a monolayer on the MOF surface (Figure 5) [33]. Further, the adsorption of the solute onto the adsorbent can be well described by a monolayer adsorption process, which assumes that only one layer of molecules can be adsorbed onto the surface of the adsorbent [59].

The values of various constants from the Langmuir adsorption model are obtained as $k_{L}$ = 0.008${Lmg}^{-1}$, 0.003 ${Lmg}^{-1}$ with a good fitting of 0.999 and 0.991 for PVC NPs and PMMA NPs, respectively (Table 2). Similarly, the values of the Freundlich constant (K_f_), empirical coefficient (n), and R^2^ obtained were 2.444 mg/g, 1.066, and 0.819 for PVC NPs and 0.222 mg/g, 1.321, and 0.845 for PMMA NPs, respectively (Table 2).

The Tempkin isotherm model shows that the adsorption behavior of the adsorbate on the surface of the adsorbent depends on the nature of the MOF-5 surface and surface charges and concentrations of PNPs at a particular temperature. Furthermore, the Temkin isotherm model determines the heat changes during the adsorption of PNPs on the MOFs surface [20,60]. However, this model gave low values for R^2^ as 0.608 and 0.772 for PVC NPs and PMMA NPs, respectively. Lastly, the Dubinin-Radushkevich (D-R) isotherm model was tested by using the adsorption data of PNPs but gave low R^2^ values of 0.607 and 0.827 for PVC NPs and PMMA NPs, respectively (Table 2).

**Figure 6:**
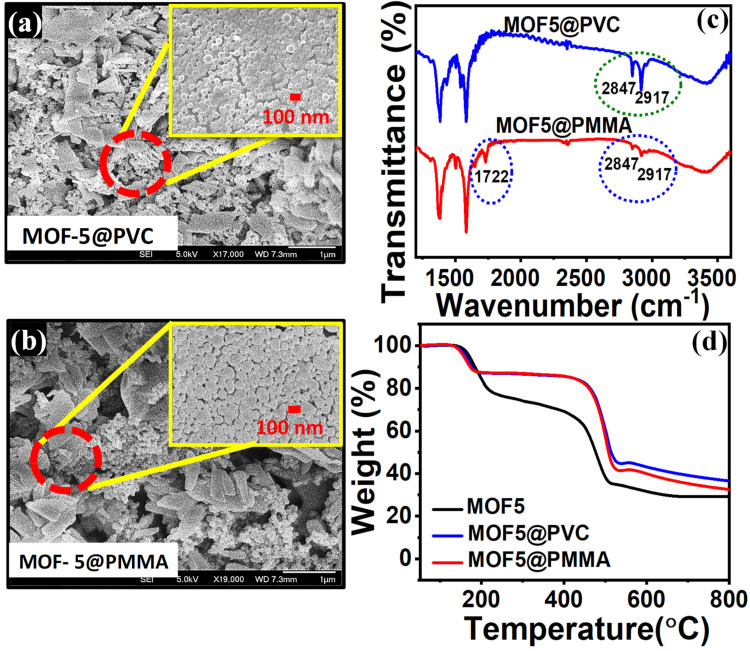
SEM images (**a**,**b**), FTIR spectra (**c**) and TGA traces (**d**) of MOF-5 after adsorption of PVC NPs and PMMA NPs. SEM images and other characterization data of the MOF before the adsorption of the PNPs are given in Figure 1. Small areas (red circle) inside the (**a**,**b**) are expanded to show the adsorption of PNPs on the MOF surface.

The SEM images of the MOF-5 after the adsorption of PVC NPs and PMMA NPs showed the presence of spherical particles on the surface (Figure 6a,b). The microscopic fluorescent images of PNP adsorbed MOF-5 showed strong green fluorescence of the PTE dye present inside the PNPs (Figure S9). In the FTIR spectra of the MOF-5 after adsorption of PNPs, the characteristic peak of the *>*C=O of the ester group present on the PMMA polymer backbone appeared at 1722 cm^−1^ and C-H groups of the PVC backbone showed strong stretching vibrations at 2917 cm^−1^ and 2847 cm^−1^ (Figure 6c) [54].

The thermogravimetric analyses of MOF adsorbed with PVC NPs and PMMA NPs were conducted between the temperature range of 25 – 900 °C. The thermal degradation patterns of the MOF-5 before and after adsorption of PNPs showed a weight loss of 17% and 23% below 200 °C considered as the loss of water or other low boiling compounds present in the MOF (Figure 6d). The higher water loss before the adsorption of PNPs indicates the high surface charge induced hydration of the MOF surface. The second weight loss of ~ 46% observed below 600 °C corresponds to the degradation of TPA and PNP contents of the MOF. Even though the degradation temperatures are the same, TGA traces of MOF after adsorption of PNPs showed a small degree of higher stability. This may be due to the oppositely charged PNPs filling the cracks on the crystal surface and acting as a physical crosslink to stabilize the MOFs. The percentage of residual mass left for all MOFs was 34% for MOF-5 @ PMMA NPs and 36% for MOF-5 @ PVC NPs at around 900 °C. The ICP analysis showed that MOF-5 contained similar amounts of Zn metal content before (18.97%) and after adsorption of PVC NPs (18.54%) and PMMA NPs (17.56%) from water (Table S1). The slight variation observed was due to added amounts of PNPs on the surface. In addition, Zn^2+^ ions were not lost or leached into water during the extraction process, and no changes in the structure of MOF were observed.

### 3.6. Regeneration of the MOF-5

**Figure 7:**
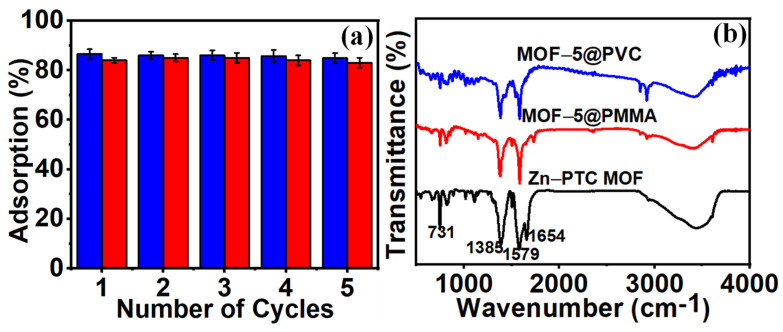
Adsorption efficiency of MOF-5 for repeated cycles of acid washing and adsorption of PVC NPs (■) and PMMA NPs (■) from water (**a**) and FTIR spectra of MOF-5 before (▬) and after adsorption of PMMA NPs (▬) and PVC NPs (▬) and washing with dil. HCl solution for regeneration (**b**).

The regeneration of the MOF-5 is used to test the reusability of the MOF, which helps to reduce wastage, lower operational costs, and enhance sustainability. Regeneration of the MOF with PNPs on the surface is achieved by washing it with strong acids or bases such as dilute HCl, HNO_3_, or NaOH solutions. However, MOF particles are unstable in strong acid or alkali media due to labile metal-ligand bonds [27]. ZIF-67 MOF was unstable in strong acid media due to the presence of labile zinc-ligand bonds [27]. The current work also observed the degradation of MOF while using concentrated HCl (0.1 M). Here, washing with dil. HCl (10 mL, 0.001 M HCl) solution was used to remove the polymer nanoparticles from the surface of MOF-5 without causing any structural damage (Figure 7a). The regenerated MOF-5 was used to extract PNPs from water solutions. During the five cycles, the observed extraction efficiencies of the regenerated MOF-5 were around 84–86% for PVC NPs and PMMA NPs. The data indicate no significant changes in the percentage of the PNP adsorption or degradation of the MOF during the five consecutive cycles of extraction and regeneration. The FTIR spectra of MOF-5 before and after adsorption of PNPs showed no major changes in the peaks, which indicates good stability even after repeated adsorption − readsorption cycles (Figure 7b).

A list of adsorbents used for the removal of plastic particles from water is presented (Table 3). The table offers a comparative analysis of various parameters, including the adsorbent utilized, types of pollutants targeted, their concentrations, and the corresponding removal efficiencies.

### 3.7. Microplastic Particle Extraction from Body Scrub Gel Using MOF-5

**Figure 8:**
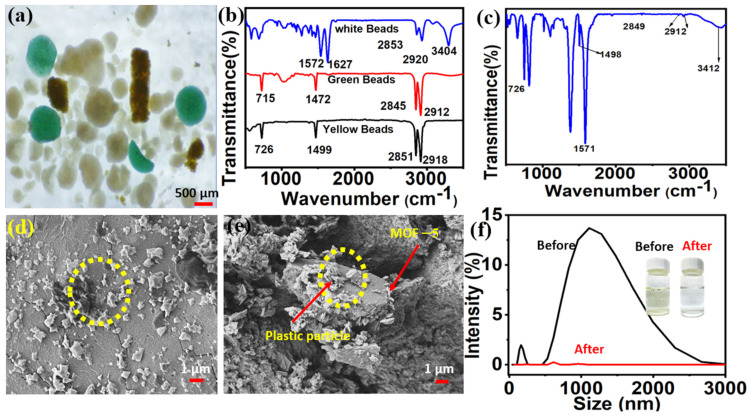
The optical image (**a**) and FTIR spectra of the colored particles before (**b**) and after adsorption on MOF-5 (**c**), SEM images microplastic particles in the scrub solution (**d**) and the surface of MOF-5 after adsorption (**e**), size distribution (**f**) of particles present in solution before (▬) and after (▬) adsorption of polymer particles on MOF-5 and optical images (inset) of body scrub solution (before and after) extraction.

Our study aimed to thoroughly investigate the extraction of microplastic particles from commercially available body scrub gel using MOF-5. The concentrated gel (1 mL) was dissolved in deionized (DI) water (50 mL), and an appropriate volume (10 mL) of the solution was stirred with MOF-5 (25 mg) for 60 min. The optical microscopic image and FTIR spectra of the MOF-5 with the plastic particles on the surface are given in Figure 8. The peak at 1498 cm^−1^ and 1571 cm^−1^ corresponds to stretching vibrations of >C=O bonds amide groups on the polymer backbone, and the peak at 726 cm^−1^ is associated with the stretching vibrations of C-O-C bonds of adsorbed particles (Figure 8c). Additionally, the FTIR spectrum of the MOF exhibited characteristic peaks at 2849 cm^−1^ and 2912 cm^−1^, corresponding to the -CH stretching vibrations. The chemical composition of the plastic particles was identified as HDPE and polyamide. A drop of the diluted body scrub solution was drop cast onto a glass slide and used for SEM imaging (Figure 8d). The average size of the plastic particles in the scrub solution before adsorption is in the range of 250–1045 nm (Figure 8d). The MOF after adsorption was also imaged using the SEM and showed the presence of plastic particles (Figure 8e). The adsorption and removal of plastic particles from the scrub solution were also monitored using DLS (Figure 8f). Particle size from the DLS measurements was found to be 1345 nm before adsorption, and no particles were detected in the solution after adsorption.

## 4. Conclusions

In summary, the MOF-5 was synthesized using the TPA recovered from the hydrolysis of PET bottle waste and used for the removal of PNPs from water. The synthesized MOF was fully characterized using various techniques such as SEM, ICP, TGA, XRD, BET analyses, and FTIR spectroscopy. The luminescent NPs of commercial PMMA and PVC polymers, with dried sizes of 95 nm and 75 nm and hydrated sizes of 189 nm and 155 nm, respectively, were prepared and used as traceable model PNPs for adsorption studies. The MOF-5, with a positive surface potential of 36.25 mV, was able to adsorb negatively charged PNPs at high efficiencies of 95% to 97%. The collected data for the adsorption of both PNPs fit well with the Langmuir adsorption isotherm model and pseudo-second-order kinetic relationship. The regeneration of the used MOF-5 was done by washing with dilute HCl solution, which showed good readsorption efficiencies of 83–85% after five cycles. The ICP data showed no leaching of Zn ions during the adsorption process, indicating high stability of MOF-5. Such easily accessible adsorbent materials are needed to mitigate the danger caused by emerging plastic pollution.

## Figures and Tables

**Table 1:** Comparison of kinetic parameters for the adsorption of PVC NPs and PMMA NPs on MOF-5.

Polymer Particles	Pseudo-First Order			Pseudo-Second Order			
	$Q_{e,calc}$ (mg/g)	$K_{1}$ (1/min)	$R^{2}$	$Q_{e,exp}$ (mg/g)	$Q_{e,calc}$ (mg/g)	$k_{2}$ (g/mg min)	$R^{2}$
PVC NP	6.615	0.479	0.972	6.555	6.702	0.039	0.996
PMMA NP	10.876	0.499	0.958	11.354	12.787	0.003	0.990

**Table 2:** Isotherm data for removing PNPs from water using MOF-5 at room temperature.

Nanoparticles		Langmuir	Freundlich	Temkin	D-R
**PVC NPs**	Adsorption Constant	K_L_ = 0.008 L/mg Q_max_ = 56.653 mg/g	K_f_ = 2.444 L/mg n = 1.066	b = 231.505 B_T_ = 10.702 J/mol	β = 4.354 × $10^{-6}$ ${mol}^{2}/{kJ}^{2}$
	$R^{2}$	0.999	0.819	0.608	0.607
**PMMA NPs**	Adsorption Constant	K_L_ = 0.003 L/mg Q_max_ = 33.324 mg/g	K_f_ = 0.2219 L/mg n = 1.321	b = 332.230 B_T_ = 7.547 J/mol	β = 1.421 × $10^{-4}$ ${mol}^{2}/{kJ}^{2}$
	$R^{2}$	0.991	0.845	0.772	0.827

**Table 3:** List of adsorbents used for the removal of plastic particles.

Adsorbent	Microplastic/ Nanoplastic	Concentration (ppm)	Removal Rate (%)	Ref.
Calcium Alginate	PET	50	>97	[61]
Coffee ground	PS	50	74	[62]
Granular AC	PS	50	98	[63]
Cr-MOF	PS	70	96	[59]
MOF-5	PVC, PMMA	250	97 & 95	This work

## Data Availability

Data are contained within the article.

## Supplementary Materials

The supporting information can be downloaded at: https://www.mdpi.com/article/10.3390/nano14030257/s1, Calibration curve for PNPs, FTIR spectra of TPA, PET and MOF, TEM and SEM images of MOF-5, kinetic and pseudo first order model for the adsorption of NPs on MOF-5, Freundlich isotherm study for the NPs adsorption in MOF-5, Temkin model isotherm studies for the adsorption of NPs on MOF-5, D-R isotherm studies foe NPs on MOF-5, fluorescent image of MOF-5 before and after adsorption. Percentage of Zn in MOF-5 before and after adsorption.
