# Supplementary material for: Conversion of PET Bottle Waste into a Terephthalic Acid-Based Metal-Organic Framework for Removing Plastic Nanoparticles from Water
Source: Nanomaterials (Basel). 2024 Jan 24;14(3):257. doi: 10.3390/nano14030257 (PMC10856359; doi:10.3390/nano14030257)
Supplement: Supplementary file 1 [file nanomaterials-14-00257-s001.zip › nanomaterials-2777041-supplementary.pdf]

Conversion of PET Bottle Waste into a Terephthalic Acid-Based Metal-Organic Framework for Removing Plastic Nanoparticles from Water

Chingakham Chinglenthoiba, Gomathi Mahadevan, Jiawei Zuo, Thiruchelvam Prathyumnan and Suresh Valiyaveettil

Department of Chemistry, National University of Singapore, 3 Science Drive 3, Singapore 117543, Singapore

Contents

Figure S1: Calibration curve for PVC (a, ■) and PMMA (b, ■) NP solutions using a concentration range of 25-250 ppm.

Figure S2: Figure S2: The FTIR spectra of PET Bottle sample (▬), commercial TPA (▬), recovered after hydrolysis of PET (▬) and MOF-5 (▬). KBr matrix was used for collecting the spectra, (1717 cm^{-1} = $-CO_2R$, 1680 cm^{-1} = $-CO_2H$, 1654 cm^{-1} = $-CO_2Zn$).

Figure S3: TEM (a), SEM (b), SEM - elemental mapping images for Zn (c) and O_2 (d) on the surface of MOF-5. N_2 adsorption-desorption isotherm curve (e, BET data) was collected after degassing the sample at 120 °C for 10 hours.

Figure S4: SEM images of the PVC (a) and PMMA (b) NPs; Size distribution (c) of PVC (▬) and PMMA (▬) NPs measured using DLS in water.

Figure S5: Kinetic studies using pseudo-first order model for the adsorption of PVC NPs (a) and PMMA NPs (b) on MOF-5 from water. The dotted line represent the line of best fit.

Figure S6: Freundlich isotherm studies for the adsorption of PVC (a) and PMMA (b) NPs on MOF-5 from water. The dotted line represent the line of best fit.

Figure S7: Temkin model isotherm studies for the adsorption of PVC (a) and PMMA (b) NPs on MOF-5 from water. The dotted line represent the line of best fit.

Figure S8: D-R isotherm model studies for the adsorption of PVC (a) and PMMA (b) NPs on MOF-5 from water. The dotted line represent the line of best fit.

Figure S9: Fluorescent microscopic images of MOF-5 (a) with no fluorescence (i.e. before) adsorption and after adsorption of luminescent PVC (b) and PMMA (c) NPs form solution. The homogenous distribution indicate strong interaction between the MOF surface and PNPs.

Citation: Chinglenthoiba, C.; Mahadevan, G.; Zuo, J.; Prathyumnan, T.; Valiyaveettil, S. Conversion of PET Bottle Waste into a Terephthalic Acid-Based Metal-Organic Framework for Removing Plastic Nanoparticles from Water. *Nanomaterials* **2024**, *14*, x. https://doi.org/10.3390/xxxxx

Academic Editor: Antonino Gulino

Received: 1 December 2023
Revised: 10 January 2024
Accepted: 15 January 2024
Published: 24 January 2024

Copyright: © 2024 by the authors. Licensee MDPI, Basel, Switzerland. This article is an open access article distributed under the terms and conditions of the Creative Commons Attribution (CC BY) license (https://creativecommons.org/licenses/by/4.0/).

Table S1: Percentage of Zn in MOF-5 before and after the adsorption of PNPs from water. Similar values indicate very little or no leaching of Zn into the water.

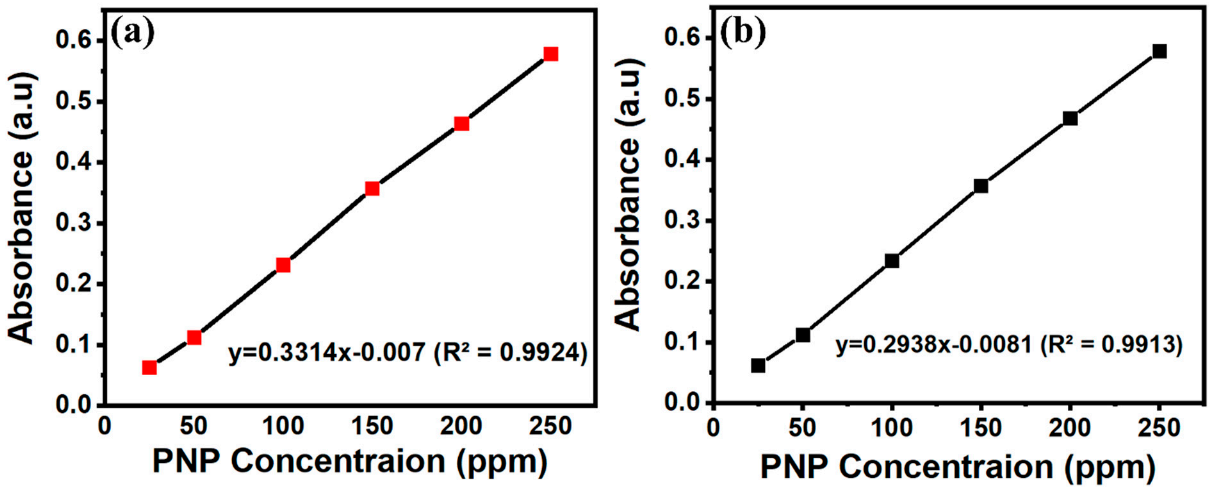

Figure S1: Calibration curve for PVC (a, ■) and PMMA (b, ■) NP solutions using a concentration range of 25-250 ppm.

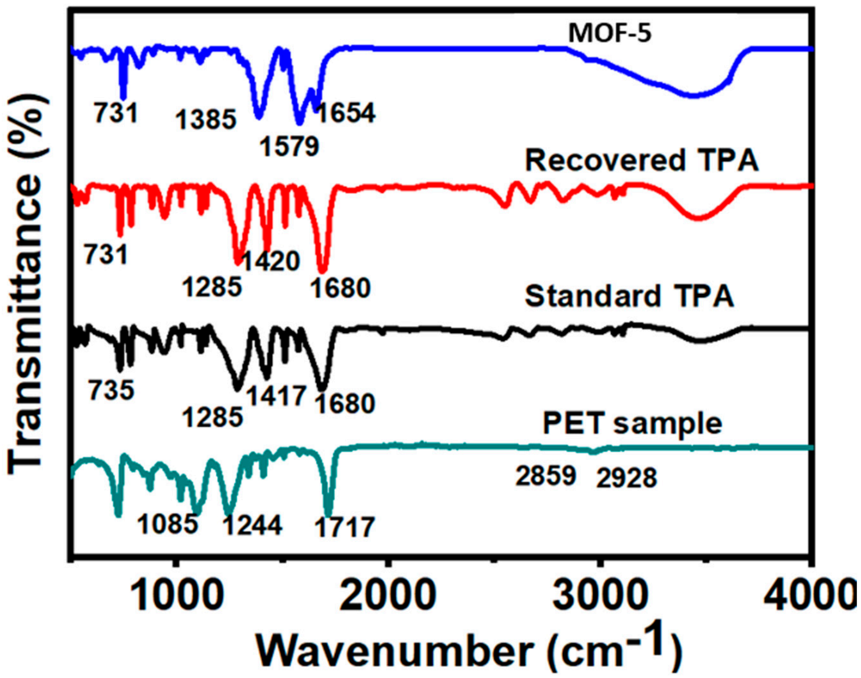

Figure S2: The FTIR spectra of PET Bottle sample (—), commercial TPA (—), recovered after hydrolysis of PET (—) and MOF-5 (—). KBr matrix was used for collecting the spectra, (1717 cm^{-1} = $-CO_2R$, 1680 cm^{-1} = $-CO_2H$, 1654 cm^{-1} = $-CO_2Zn$).
